# Effectiveness and Safety of Different Estradiol Regimens in Transgender Women (TREAT Study): Protocol for a Randomized Controlled Trial

**DOI:** 10.2196/53092

**Published:** 2023-12-22

**Authors:** Samuel Cortez, Dominic Moog, Christopher Lewis, Kelley Williams, Cynthia Herrick, Melanie Fields, Teddi Gray, Zhaohua Guo, Ginger Nicol, Thomas Baranski

**Affiliations:** 1 Division of Pediatric Endrocinology Department of Pediatrics Washington University School of Medicine St Louis, MO United States; 2 Washington University School of Medicine St Louis, MO United States; 3 Division of Endocrinology, Diabetes, and Lipid Metabolism Department of Medicine Washington University School of Medicine St Louis, MO United States; 4 Division of Public Health Sciences Department of Surgery Washington University School of Medicine St Louis, MO United States; 5 Division of Pediatric Hematology/Oncology Department of Pediatrics Washington University School of Medicine St Louis, MO United States; 6 Department of Neurology Washington University School of Medicine St Louis, MO United States; 7 Department of Psychiatry Washington University School of Medicine St Louis, MO United States

**Keywords:** transgender research, transgender health, clinical trial, estrogen, transwomen

## Abstract

**Background:**

Current guidelines for gender-affirming hormone therapy (GAHT) for transgender women are mostly based on clinical experience from experts in the field and treatments used on postmenopausal women. While care is currently provided with the best available evidence, there is a critical gap in knowledge about the safest and most effective estradiol routes of administration for GAHT in transgender women; this statement is supported by the World Professional Association for Transgender Health on their *Standards of Care for the Health of Transgender and Gender Diverse People, version 8*. Furthermore, the reported rates of cardiometabolic adverse events in transgender women highlight the importance of investigating changes in lipoproteins, glucose, and insulin sensitivity, among other markers while receiving GAHT.

**Objective:**

This study aims to evaluate the degree of testosterone suppression achieved at 1, 6, and 12 months in treatment-naive transgender women when randomized to GAHT with estradiol and spironolactone as antiandrogens. As a secondary aim, this study will assess the treatment effect on metabolic and coagulation factors from baseline to 6 and 12 months after initiating GAHT.

**Methods:**

This is a prospective pilot, open-label, randomized clinical trial conducted at an adult transgender clinic in a tertiary medical center. The 3 treatment arms include once-daily sublingual 17-β estradiol, twice-daily sublingual 17-β estradiol, and transdermal 17-β estradiol. All participants received spironolactone as an antiandrogen. Transgender women aged 18 to 45 years who are being evaluated for the initiation of GAHT with 17-β estradiol and did not have a history of coagulopathy, cigarette smoking, liver disease, dyslipidemia requiring treatment, or use of gonadotropin-releasing hormone agonist were eligible to enroll. The main outcome is the total testosterone suppression at 1 and 6 months after the initiation of GAHT, and the secondary outcome is to assess treatment effect in a lipid panel; homeostatic model assessment for insulin resistance; coagulation factors II, IX, and XI; Von Willebrand factor; activated protein C resistance; protein C; and protein S at baseline, 6 months, and 12 months after therapy is initiated.

**Results:**

This study was funded in March 2022, and enrollment concluded in August 2022. It was concluded in July 2023, and currently, the results are being analyzed for publication.

**Conclusions:**

The Transgender Estradiol Affirming Therapy (TREAT) study offers a rigorous and reproducible approach to answer important questions regarding GAHT in transgender women, specifically, the most effective 17-β estradiol regimen to suppress testosterone levels to 50 ng/dL, as currently recommended.

**Trial Registration:**

ClinicalTrials.gov NCT05010707; https://clinicaltrials.gov/study/NCT05010707

**International Registered Report Identifier (IRRID):**

DERR1-10.2196/53092

## Introduction

Transgender individuals experience discordance between their self-identified gender and biological birth sex [1-3]. Some transgender individuals experience gender dysphoria, which the *Diagnostic and Statistical Manual of Mental Disorders* defines as clinically significant distress or impairment in social, occupational, or other key areas of functioning. Increasing numbers of adolescents seek care at gender identity centers in Western countries [4,5].

Studies using short self-reports of gender identity and its variants suggest that 0.17% to 1.3% of adolescents and young adults identify as transgender [6]. Even using the conservative estimate of 0.3%, the number of people living in the United States who identify as transgender is nearly 1 million [7]. Given the widespread acknowledgment of the health care needs of this population, priority should be given to those actions that will ensure timely access to appropriate care. Such action includes coverage of transition care and a requirement to ensure safe, effective, appropriate, and sensitive care in health centers [8-10].

Transgender women seek gender-affirming hormone therapy (GAHT) to reach physical features consistent with their gender identity [11-13]. The goal of GAHT for transgender women is to achieve feminine features and maintain estradiol levels within the normal woman range while suppressing endogenous testosterone [11]. The current approach to GAHT is not uniform and is used off-label for this indication. It also depends on the cost of the regional health care system and availability of estrogens and antiandrogens.

A typical regimen includes estrogen to provide feminizing effects in conjunction with therapy to block testosterone (antiandrogens or gonadotropin-releasing hormone analogs) [14]. The currently preferred estrogen formulation that can be provided in tablets, patches, and injections is 17-β estradiol, and spironolactone is the most prescribed antiandrogen in the United States.

Studies have demonstrated that transgender individuals on hormone therapy have higher rates of adverse cardiovascular events (myocardial infarction, venous thromboembolism, and ischemic stroke), highlighting the importance of investigating surrogate markers such as changes in lipoproteins, estrone, glucose, and insulin-sensitivity based on the estrogen preparation used during GAHT [8,15-21].

Both the World Professional Association for Transgender Health and the Endocrine Society have created transgender-specific guidelines that serve as a framework for providers caring for gender minority patients [9,22]. These guidelines are primarily based on clinical experience from experts, and guidelines for estrogen therapy in transgender women are loosely based on treatments for postmenopausal women [1]. Therefore, stronger evidence is needed to guide clinicians on best clinical practices for this population, as stated in the *Standards of Care for the Health of Transgender and Gender Diverse People, version 8*: “Determining the safest and most efficacious estrogen compound and route of administration for transgender people is an important topic” [9,22,23].

For this proposed study, we aimed to evaluate the degree of testosterone suppression and treatment-related changes in metabolic and coagulation factors in transgender woman patients undergoing randomized GAHT for a period of 12 months. Participants were randomized to once-daily sublingual 17-β estradiol, twice-daily sublingual 17-β estradiol, or transdermal 17-β estradiol, using spironolactone as an antiandrogen in all 3 treatment arms.

We chose to look at once versus twice-daily sublingual 17-β estradiol to evaluate if there is a difference in the level of suppression of gonadotrophins based on 1 larger peak serum level of estradiol versus 2 peaks. To date, no studies have looked at the effects of estradiol pulses (once vs twice) on suppression of the hypothalamic or pituitary axis.

## Methods

### Overview

This prospective pilot, open-label, randomized clinical trial compares the testosterone-suppressing effects of 3 commonly used 17-β estradiol regimens in transgender woman adults initiating GAHT. The 17-β estradiol regimens used in this trial include once-daily sublingual 17-β estradiol, twice-daily sublingual 17-β estradiol, and transdermal 17-β estradiol patch.

The primary end point is testosterone suppression from baseline to 1 and 6 months with secondary evaluation of treatment-related changes in lipid panel; homeostatic model assessment for insulin resistance; coagulation factors II, IX, and XI; Von Willebrand factor; activated protein C resistance; protein C; and protein S at baseline, 6 months, and 12 months after therapy is initiated.

The study took place at the Washington University Transgender Center. Patients aged 18 to 45 years who were being evaluated for the initiation of GAHT with 17-β estradiol and did not have a history of coagulopathy, cigarette smoking, liver disease, dyslipidemia requiring treatment, or use of gonadotropin-releasing hormone agonist were eligible to enroll. Initially, the study was limited to those aged 18-30 years and with BMI <30 kg/m^2^, but these were later removed to increase the population available to screen and participate in the study.

Power analyses indicated that 12 participants per group would provide 80% power to detect differences in total testosterone between groups when the true difference between groups is 230 ng/dL. The type I error probability is .05.

We will use a likelihood-based mixed-effects model to separately evaluate the effects of time and treatment assignment on serum testosterone, estradiol, and estrone from baseline to 1 and 6 months after initiation of medications.

We will also use a likelihood-based mixed-effects model to separately evaluate the effects of time and treatment on metabolic and coagulation factors from baseline to 6 and 12 months after the initiation of treatment. We will also determine if there is any correlation between estradiol and estrone levels with coagulation factors as suggested by other studies.

### Ethical Considerations

The Washington University in St. Louis Institutional Review Board approved the study protocol (IRB# 202104092) in May 2021, which was executed in accordance with the Declaration of Helsinki. Participants received financial compensation for their time and costs associated with participation in the study ($30 per visit).

### Study Procedures

Participants who met eligibility criteria and provided informed consent were randomized 1:1:1 to either once-daily sublingual 17-β estradiol, twice-daily sublingual 17-β estradiol, or a transdermal 17-β estradiol patch. All participants received spironolactone as an antiandrogen. The dosing protocol is shown in Table 1.

**Table 1:** Dosing regimen per treatment group.

Medication	Once-daily sublingual	Twice-daily sublingual	Transdermal
17-β estradiol	Starting dose: 2 mg once a day Increase by 2 mg/day	Starting dose: 1 mg twice daily Increase by 2 mg/day	Starting dose: 100 mcg/24 h Increase by 100 mcg/24 h
Spironolactone	Starting dose: 50 mg daily Increase by 50 mg/day Maximum dose: 200 mg/day	Starting dose: 50 mg daily Increase by 50 mg/day Maximum dose: 200 mg/day	Starting dose: 50 mg daily Increase by 50 mg/day Maximum dose: 200 mg/day

Initial evaluation included a comprehensive medical history, physical examination, and baseline laboratory testing. During the initial visit, patients were randomized and started on spironolactone and 17-β estradiol based on their treatment group assignment. The initial evaluation was completed in conjunction with the treating endocrinologist with follow-up conducted by the study investigator.

Once participants started their GAHT treatment assignment, they underwent a titration phase in which the hormone profile including estradiol, total testosterone, estrone, and basic metabolic panel was assessed every 4 weeks until total testosterone was less than 50 mg/dL. If the total testosterone is above goal, 17-β estradiol and spironolactone doses were adjusted based on their group dosing regimens as depicted in Table 1.

Anthropometric measurements and metabolic and coagulation factors were obtained at baseline, 24 weeks, and 52 weeks (Table 2). Blood testing was conducted before their morning dose for participants randomized to sublingual 17-β estradiol or the morning when the transdermal 17-β estradiol patch is due for replacement.

**Table 2:** Study visits and laboratory testing.

Laboratory testing			Baseline		Titration phase	6 months		12 months
**Anthropometric measurements**								
	Weight	✓				✓	✓	
	Height	✓				✓	✓	
	BMI	✓				✓	✓	
	Waist circumference	✓				✓	✓	
	Blood pressure	✓				✓	✓	
**Hormone profile**								
	Total testosterone	✓		✓		✓	✓	
	Estradiol	✓		✓		✓	✓	
	Estrone	✓		✓		✓	✓	
Basic metabolic panel			✓		✓	✓		✓
Liver function panel			✓			✓		✓
**Metabolic parameters**								
	Fasting serum glucose	✓				✓	✓	
	Fasting total insulin	✓				✓	✓	
	Fasting lipid panel	✓				✓	✓	
**Coagulation factors**								
	Factor II activity	✓				✓	✓	
	Factor IX activity	✓				✓	✓	
	Factor XI activity	✓				✓	✓	
	Von Willebrand Factor Antigen	✓				✓	✓	
	Activated protein C resistance (APCR)	✓				✓	✓	
	Protein C activity	✓				✓	✓	
	Free protein S antigen	✓				✓	✓	

## Results

This study was funded in March 2022, and enrollment concluded in August 2022. It was concluded in July 2023, and currently, the results are being analyzed for publication.

## Discussion

### Expected Findings

The Transgender Estradiol Affirming Therapy (TREAT) study offers a rigorous and reproducible approach to answer important questions regarding the GAHT in transgender women. Specifically, the study will determine if significant differences in testosterone suppression between once-daily, twice-daily, or transdermal 17-β estradiol exist.

This study will also provide insights into metabolic and coagulation changes in transgender women on GAHT with sublingual and transdermal 17-β estradiol and spironolactone as antiandrogens. These outcomes, with the GAHT used in this study, have not been explored in prior studies.

In addition, this study will provide insights into the best strategies and most reliable methods to inform the design of future, fully powered randomized clinical trials.

### Limitations

Due to a lack of uniformity on the current prescription patterns for GAHT, we decided to conduct this study using the most common regimens prescribed in our clinical setting with no prior studies describing their effects on metabolic and coagulation factors. Hence, it may not be representative of other approaches to GAHT in transgender women, including estradiol valerate or cypionate, which were not included in the study.

### Conclusions

The proposed study addresses areas of research that have been determined as important by the most recent World Professional Association for *Standards of Care for the Health of Transgender and Gender Diverse People, version 8*, including the most effective estrogen regimen (route of administration and dosing schedule), while providing insights on metabolic and coagulation factor changes with concurrent use of spironolactone, which has not been explored previously. Furthermore, this study provides insight into methodology and statistics that will inform larger clinical trials.

## Appendix

Multimedia Appendix 1 Peer reviewe report from Washington University Institute of Clinical and Translational Sciences.

Multimedia Appendix 2 CONSORT checklist.

## Abbreviations

GAHT: gender-affirming hormone therapy
TREAT: Transgender Estradiol Affirming Therapy
