# Supplementary material for: Effectiveness and Safety of Different Estradiol Regimens in Transgender Women (TREAT Study): Protocol for a Randomized Controlled Trial
Source: JMIR Res Protoc. 2023 Dec 22;12:e53092. doi: 10.2196/53092 (PMC10770781; doi:10.2196/53092)
Supplement: Multimedia Appendix 1 [file resprot_v12i1e53092_app1.pdf]

Washington University Institute of Clinical and Translational Sciences
Clinical and Translational Research Funding Program
Summary of Reviewer Comments
(Confidential)

ICTS Program Submission Cycle – September 2021 **Summary Statement Issue Date** – November 2021
Principal Investigator: Baranski, Thomas
Project Title: Effectiveness, Safety, and Tolerability of Different Estradiol Dosing Regimens in Transgender Females

Overall Project Evaluation:

After full consideration by the review committee, this application was considered to be in the Outstanding range.

Strengths

Reviewer 1:

- This is a feasibility study that aims to study dosing regimens and safety profiles of estrogen formulations in transgender females. This is an understudied population and the field certainly needs more evidence-based guidance.
- Excellent preliminary data assessing patient interest in participation and thus feasibility of study completion.
- Detailed 2 year evaluation of anthropomorphic and laboratory testing in order to gather preliminary data in order to guide data collection in future larger studies.

Reviewer 2:

- This study addresses an important problem in a marginalized and understudied population (i.e., the lack of research on gender affirming hormone therapy represents a critical gap in knowledge that precludes the delivery of safe and appropriate transgender care.)
- Results from this research will provide prelim data for studies that can generate evidence and inform standards in clinical care. This is highly significant work.
- The research compares three treatments which are administered regularly, despite inadequate evidence.
- The PI serves as the Co-Director of the Transgender Center at Wash U, positioning him perfectly for this research. Dr. Nicol (Co-I) has extensive experience conducting clinical trials studying pharmacotherapies in youth and young adults.
- The approach clearly spells out inclusion and exclusion criteria, the experimental design, and the protocol.
- The scientific environment is excellent.

Reviewer 3:

- Addresses critical disparities in care. In the absence of preliminary data, the qualitative patient engagement data adds to the feasibility of the proposed study.

Weaknesses

Reviewer 1:

- There is extensive laboratory evaluation every 6 months. Only some of these are included in the budget. I assume that the rest are standard of care and are part of the clinical evaluation, but some clarification on which tests are experimental and which are standard of care would benefit the reader.
- There is a paucity of preliminary data, although this is not necessarily a weakness in the setting of a feasibility study and is certainly addressed by the PI.

Reviewer 2:

- The requested study period is wrong...beginning date is 3/31/22 and not 8/3/21. The application describes that the project will take longer than one year (the study period).
- Proposal has several typos and appears spellcheck not used.

- Dr. Nicol's biosketch says that this research will support "the career development of a promising physician-scientist in pediatric endocrinology, Samuel Cortez," yet Dr. Cortez was not mentioned anywhere else on the application or budget.
- The authors seem to use the terms efficacy and effectiveness interchangeably. Is this an efficacy trial determining results under ideal circumstances? Or an effectiveness trial - or pragmatic trial - measuring the degree of beneficial effect under real-world conditions? The authors need to choose one and stick with it.
- The application is titled "Effectiveness, safety, and tolerability of different estradiol dosing regimens in transgender females" yet the investigators do not spell out what measures are effectiveness, safety, or tolerability. They could connect the dots for the reviewer a bit more clearly.

Reviewer 3:

- Aims seem feasible in 1-year, but proposed timeline is 2 years. It would be helpful to better describe the purpose for the additional year of follow-up. Will enough data be collected within 1-year to support next stage funding?

Recommendations for Improvement

Reviewer 1:

- Consider assessing patient satisfaction and tolerability/side effect profiles with regimens as well. Although this may be more relevant as an outcome in a larger RCT, survey administration feasibility is also important to address.

Reviewer 2:

- See weaknesses section

Reviewer 3:

ICTS BIOSTATISTICS, EPIDEMIOLOGY AND RESEARCH DESIGN (BERD) Review Comments

- Weaknesses:
  I have moderate epidemiological or biostatistical concerns for this study.
  The randomized feasibility trial appears to be well-designed. As a minor point, the exclusion criteria are fairly restrictive (i.e., no smokers or obese participants), but this may be appropriate at this stage of investigation.
  As moderate methodologic concerns, the trial is described as a two-year study, but only data from the 6-month follow-up visit will be available on all or most participants during the 1-year ICTS grant period, assuming that it takes <6 months to recruit all participants. In addition, the outcome measures of the trial were not clearly described. For Aim 1, I assume that the outcome will be testosterone concentration (or possibly % testosterone suppression) at the 6-month follow-up visit, but this was not clear. For Aim 2, I assume that the outcomes will be concentrations of estrone, thrombogenic factors, and differences between concentrations of baseline and 6-month metabolic factors. Finally, the rationale for the sample size was not described - i.e., what does the study have the appropriate sample size to do? Estimate a sufficiently precise measure of feasibility? Generate preliminary estimates of efficacy that would be useful to inform the sample size for a future, sufficiently powered trial? Without this information, it is difficult to understand the likely utility of the study.

Health equity comments (if applicable)

- The research team should ensure recruitment efforts include outreach to Black and other minority transgender females so that the study does not result in bias towards only white transgender females.

9-Point Score Chart

Impact	Score	Descriptor	Guidance
High Impact	1	Exceptional	Exceptionally strong with essentially no weaknesses
	2	Outstanding	Extremely strong with negligible weaknesses
	3	Excellent	Very strong with only some minor weaknesses
Moderate Impact	4	Very Good	Strong but with numerous minor weaknesses
	5	Good	Strong but with at least one moderate weakness
	6	Satisfactory	Some strengths but also some moderate weaknesses
Low Impact	7	Fair	Some strengths but with at least one major weakness
	8	Marginal	A few strengths and a few major weaknesses
	9	Poor	Very few strengths and numerous major weaknesses
